# De-implementation strategy to reduce overtreatment of asymptomatic bacteriuria in the emergency department: a stepped-wedge cluster randomised trial

**DOI:** 10.1177/20499361241293687

**Published:** 2024-12-14

**Authors:** Tessa M.Z.X.K. van Horrik, Bart J. Laan, Janneke E. Stalenhoef, Cees van Nieuwkoop, Joppe B. Saanen, Caroline Schneeberger, Eefje Jong, Suzanne E. Geerlings

**Affiliations:** Internal Medicine, Infectious Diseases, Amsterdam UMC, University of Amsterdam, Amsterdam, the Netherlands; Amsterdam Institute for Infection and Immunity, Amsterdam Public Health, Meibergdreef 9, Amsterdam, North-Holland 1105 AZ, the Netherlands; Internal Medicine, Infectious Diseases, Amsterdam UMC, University of Amsterdam, Amsterdam, the Netherlands; Amsterdam Institute for Infection and Immunity, Amsterdam Public Health, Amsterdam, North-Holland, the Netherlands; Internal Medicine, Dijklander Hospital, Hoorn, North-Holland, the Netherlands; Internal Medicine, Infectious Diseases, OLVG, Amsterdam, North-Holland, the Netherlands; Internal Medicine, Haga Teaching Hospital, South-Holland, the Netherlands; Emergency Medicine, Amsterdam UMC, University of Amsterdam, Amsterdam, North-Holland, the Netherlands; Centre for Infectious Disease Control, National Institute for Public Health and the Environment, Utrecht, the Netherlands; Internal Medicine, Meander Medical Centre, Utrecht, the Netherlands; Internal Medicine, Infectious Diseases, Amsterdam UMC, University of Amsterdam, Amsterdam, the Netherlands; Amsterdam Institute for Infection and Immunity, Amsterdam Public Health, Amsterdam, North-Holland, the Netherlands

**Keywords:** asymptomatic bacteriuria, antimicrobial stewardship, quality improvement, urinary tract infections

## Abstract

**Background:**

Asymptomatic bacteriuria (ASB) is the presence of bacteria in the urine of patients without symptoms of a urinary tract infection. Generally, treating ASB is not beneficial.

**Objective:**

We aimed to reduce overtreatment of ASB in the emergency department (ED) through a multifaceted de-implementation strategy.

**Design:**

A stepped-wedge cluster randomised trial.

**Methods:**

We performed our study in five EDs in the Netherlands from December 2020 to December 2021. Adult patients with urine cultures obtained during ED presentation were screened for inclusion and we excluded patients with indications for antibiotic therapy. The de-implementation strategy included education, reminders and competitive feedback on baseline results. The primary endpoint was patients with ASB treated with antibiotics. Secondary endpoints included the treatment duration and the number of urine tests ordered (urinalyses and urine cultures) in the ED per 1000 adult patients.

**Results:**

In total, 6837 urine cultures were screened. ASB was present in 224/3289 (7%) and 201/3548 (6%) patients, from whom 65/224 (29%) and 46/201 (23%) were inappropriately treated with antibiotics in the baseline and intervention period, respectively (adjusted odds ratio 1.20, 95% CI 0.56–2.62, *p* = 0.65). The number of urinalyses ordered decreased from 182 to 153 per 1000 patients (incidence rate difference −29.10, 95% CI −46.36 to −11.78, *p* < 0.001). Further, the treatment duration was shortened for patients with ASB in the intervention period (baseline period: 7.98 days (standard deviation (SD) 4.31) vs 5.79 days (SD 3.33), *p* = 0.006).

**Conclusion:**

Diagnostic stewardship by our de-implementation strategy reduced the number of urinalyses ordered and treatment duration in the ED, but we found no significant reduction in overtreatment of ASB.

**Trial registration:**

The trial was registered at https://onderzoekmetmensen.nl/en/trial/25918, on 17-12-2019, registration number NL8242. The first participants were enrolled on 01-12-2020.

## Plain language summary

A de-implementation strategy to reduce overtreatment of asymptomatic bacteriuria

In this article, we describe the results of our multifaceted de-implementation strategy we used to reduce overtreatment of asymptomatic bacteriuria (ASB). ASB is the presence of bacteria in the urine of a patient who does not have symptoms of a urinary tract infection (UTI). Antibiotic treatment of ASB is discouraged by international guidelines, since this does not lead to benefits. Inappropriate urine testing contributes to overtreatment of ASB. Therefore, diagnostic stewardship focuses on improving appropriate urine testing as part of antimicrobial stewardship. The de-implementation strategy consisted of education and reminders about appropriate urine testing, and treatment of ASB, and feedback about the baseline period. We introduced this strategy in the emergency departments of five hospitals in the Netherlands. After the introduction of our strategy, the percentage of overtreatment of ASB was not reduced. However, the number of urinalyses ordered was reduced, along with the duration of inappropriate antibiotic treatment. We concluded that the results indicate that multiple steps in diagnostic stewardship are necessary to reduce overtreatment of ASB.

## Background

Asymptomatic bacteriuria (ASB) is the presence of bacteria in the urine of patients without symptoms of a urinary tract infection (UTI). According to clinical guidelines, ASB does not require treatment with antibiotics in most patients,^1^ but is still frequently inappropriately overtreated.^2^ The American Choosing Wisely campaign listed inappropriate treatment of ASB as one of five things physicians and patients should question,^3^ as this contributes to the increase of antimicrobial resistance.^4^ Subsequently, the campaign “To do or not to do?” was initiated to de-implement low-value care in the Netherlands.^5^

Urine tests are often ordered in the emergency department (ED) for various clinical indications,^6^ contributing to inappropriately diagnosing the presence of bacteria in urine as UTIs and overtreatment of ASB.^7^ Factors associated with inappropriate urine testing are the favourable characteristics of urine tests (e.g., cheap and non-invasive), and time pressure.^8^ Additionally, fear of a complicated disease course, time pressure, overcautiousness, insecurity, and positive urinalysis are associated with inappropriate treatment of ASB.^9–11^ In 2020, we published the results of a quality improvement study in the Netherlands and showed that most urine tests of patients admitted to the internal medicine and non-surgical subspecialty wards were ordered during ED presentation.^12^ In this study, 35% of urine cultures and 56% of microscopic urinalyses were performed unnecessarily after a negative urinalysis result.^12^ Further, the results of a recently performed American quality improvement study showed that strategies to reduce unnecessary urine cultures through diagnostic stewardship result in a reduction of ASB-related antibiotic use.^13^ However, most clinical decisions regarding urine testing in EDs are based on the urinalysis result and these tests were not included in this recent stewardship initiative by Vaughn et al.^13^ Therefore, we conducted a quality improvement project to reduce the overtreatment of ASB by using a multifaceted de-implementation strategy in five EDs in the Netherlands.^14^

## Methods

### Study design and setting

**Figure 1.**
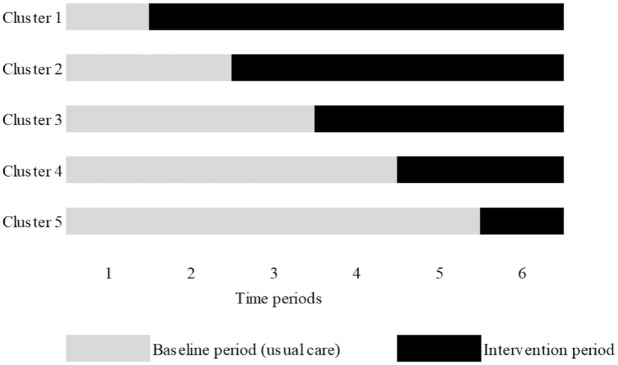
The stepped-wedge design.

A stepped-wedge cluster randomised trial in the ED of five hospitals (clusters), including one university hospital and four general teaching hospitals, in the Netherlands from December 2020 to December 2021. Each period consisted of 2 months, leading to a total study duration of 12 months (Figure 1). The order in which the de-implementation strategy was introduced in the hospitals, was randomly determined and the participating hospitals were informed before the intervention period started. In the Netherlands, physicians of different medical specialties work in the ED. Therefore, we reported the treating medical specialties of the patients and used this information in the feedback reports to the hospitals. We used the CONSORT 2010 extended checklist for the reporting of cluster randomised trials reporting the results (Supplemental File 2).^15^

### Study definitions

We defined ASB as the growth of one or more species of bacteria in quantitative counts of ⩾10^5^ CFU/mL in a urine culture collected from a patient without UTI-related signs or symptoms.^1^ We considered the following symptoms indicative of UTIs: fever, suprapubic tenderness, costovertebral angle pain or tenderness with no other recognised cause, urinary urgency, urinary frequency or dysuria.^16^

We defined a positive urinalysis as a positive dipstick for nitrite and/or leukocyte esterase, or the presence of leukocytes and/or bacteria in microscopic analysis, according to local standard operating protocols in the laboratories.^17^

Since urine culture results are not immediately available upon ED presentation, immediate clinical decisions regarding urine tests rely on the urinalysis results. Urine culture results are most likely preceded by positive urinalyses, that are associated with inappropriate antibiotic treatment.^9^ Therefore, we defined an additional group of asymptomatic patients with contaminated urine cultures in this study. These contaminated urine cultures are defined as growth of ⩾10^3^ colony-forming units (CFU)/mL of any microorganism, both uropathogens and non-uropathogens, mixed growth, including urine cultures marked as contaminated by the clinical microbiologist. Negative urine cultures were defined as urine cultures without any growth.

### Selection of participants

We screened all adult patients who visited the EDs from whom a urine culture was obtained for inclusion. We excluded urine cultures without any growth. All positive and contaminated urine cultures were screened for inclusion. Additionally, we screened all adult patients in the EDs from whom urinalyses were obtained in the first study month to investigate inappropriate antibiotic treatment of patients with a positive urinalysis result without UTI-related symptoms.

We excluded patients with treatment indications for ASB (i.e., pregnancy, neutrophil count <500 cells/µL and invasive urological procedures associated with mucosal trauma).^1^ Other exclusion criteria were active treatment for UTI on ED presentation and an alternative site of infection for which antibiotics were prescribed. An acutely altered mental status or confusion, delirium, and falling were regarded as systemic symptoms of UTIs if these patients had bacteriuria and no other foci of infections.

### Measurements

We obtained lists containing urine test results from the clinical chemistry and microbiology laboratories. Data were collected in Castor EDC.^18^ The diagnostic and therapeutic management of the urine test results were evaluated by retrospective chart review by one research physician (TMZXKvH), who was not blinded. The electronic medical files documentation upon ED presentation were screened for the presence of UTI-related symptoms and other signs of infections, including vital parameters, according to the definitions of ASB and UTI.^1,16^ We considered symptoms absent if these were not reported in the medical file. If the diagnosis of ASB or the clinical indication for antibiotics was uncertain, this was discussed with two members of the project team (SEG and BJL) until a consensus was reached. A random sample containing 10% of the data was audited by the local champion or a physician of the research team to assess the completeness and accuracy of the data and did not result in adjustments of the primary outcome. Full details are in our study protocol.^17^

### Interventions: De-implementation strategy

Through our de-implementation strategy, we distributed knowledge and raised awareness about ASB and urine testing. Each intervention period started with the general part and a local part (Table 1).

**Table 1.** Description of the de-implementation strategy.

Item	Description
**General part**	A kick-off meeting during which the baseline measurements per hospital were presented as competitive feedback and distribution of pocket cards (Supplemental Figure 1), posters and newsletters to relevant medical specialties and clinicians working in EDs. Further, we organised educational meetings for emergency physicians and nurses in all hospitals. In the Netherlands, physicians of many medical specialties (e.g., internal medicine, geriatrics, neurology and surgery) work in the ED, therefore, we organised educational meetings in these specialties. During all meetings, the value of the clinical assessment of a patient about the consideration of the urine test results was highlighted through the use of exemplary patient cases, and the diagnostic and treatment indications for urine testing (both urinalysis and urine cultures) were discussed.
**Local part**	In each hospital, we appointed a local champion, who was an internal medicine physician and/or a member of the antimicrobial stewardship team (A-team). The local champion set up an ASB team together with a resident internal medicine and was responsible for the local part of the de-implementation strategy. For example, through evaluating diagnostic and therapeutic management of positive urine test results during daily or weekly patient rounds or meetings/reports.
**Additional strategy components**	Depended on the baseline measurement feedback. For example, laboratory order set adjustments.

### Outcomes

The primary outcome was the proportion of patients with ASB treated before and after the de-implementation strategy. Secondary outcomes were the asymptomatic patients with contaminated urine cultures who received antibiotic treatment, the number of urinalyses and urine cultures ordered per 1000 patients in the ED, treatment duration for ASB and contaminated urine cultures, and the percentage of inappropriate treatment of asymptomatic patients with positive urinalyses in the first study month. We collected data about the antibiotic prescription duration of both admitted patients and patients discharged from the ED.

### Analysis

We calculated our sample size using results of previous studies that showed a 50% reduction of overtreatment of ASB from 0.450 to 0.225 following de-implementation strategies.^2^ Assuming an intra-cluster correlation coefficient of 0.10, we needed at least 420 patients (=14 patients * six periods * five hospitals) to achieve 80% power to detect this reduction using the two-sided Wald *Z*-test at a 0.05 significance level.

We used descriptive statistics to summarise the categorical data. We obtained odds ratios (ORs), adjusted for clustering and temporal trends by including the clusters (hospitals) as a categorical predictor, and the time in months as a fixed effect in the logistic regression models to analyse the effect of the de-implementation strategy.^19^ We included confounders in the model if they had a significant univariate effect (*p* < 0.1) using forward selection and selected variables based on risk factors and outcomes associated with overtreatment of ASB.^9^ The differences between the baseline period and intervention period were evaluated using an intention to treat principle. We used Akaike’s information criterion (AIC) and analysis of variance tables to compare the models and report adjusted, unadjusted, and intercept models. A two-sided *p*-value of <0.05 was considered statistically significant.

Incidence ratios (IRs) per 1000 adult patients per study period were calculated for the number of urinalysis and urine cultures ordered. We used linear regression models to analyse the incidence rate differences (IRD) and the overall effect of the de-implementation strategy on the number of urine tests ordered. We used *t*-tests or chi-square tests where appropriate for explorative subgroup analyses. We used IBM SPSS Statistics version 28.0, Armonk, NY, and RStudio version 1.3.1093, PBC, Boston, MA, for the analyses.

### Process and economic evaluation

We evaluated the de-implementation strategy adherence and compliance by assessing the numbers and types of implemented strategy components of the de-implementation strategy per hospital. We performed an explorative budget-impact analysis to evaluate the cost-effectiveness. Study materials and salary of the primary researcher to provide kick-off and educational meetings represented the costs of the de-implementation strategy. We regarded the time invested by the local champions and other healthcare professionals as part of their daily work. The number of urine tests ordered represented the main cost benefits due to our strategy. The costs for urinalyses (€5,53) were based on the Dutch Healthcare Authority.^20^ Based on the IRD of urinalysis ordered per 1000 patients in the intervention period, we calculated the hypothetical number of urinalyses ordered in the study period. We estimated the costs for maintaining the de-implementation strategy based on a monthly screening of the number of urine tests ordered in one hospital by an antimicrobial stewardship specialist.

## Results

### Characteristics of study subjects

**Figure 2.**
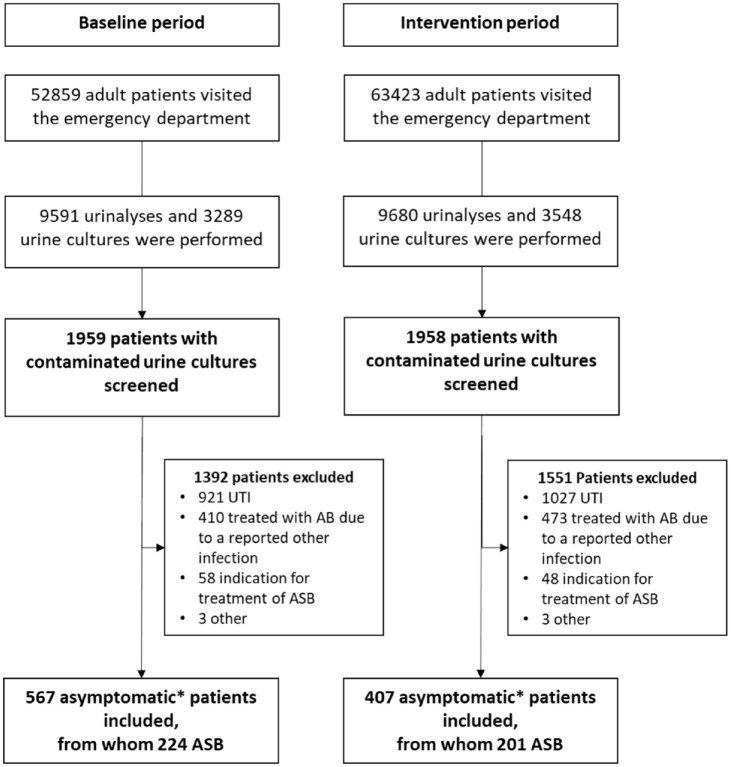
Flowchart of included patients. *Patients without UTI-related symptoms, including patients with other foci of infections for which antibiotics were not indicated according to clinical guidelines. AB, antibiotics; ASB, asymptomatic bacteriuria; UTI, urinary tract infection.

In total, 116,282 adult patients visited the EDs, from whom 19,271 urinalyses and 6837 urine cultures were obtained in five hospitals during the study period (Supplemental Table 2). In total, 813/974 (84%) urine cultures were preceded by a positive urinalysis result. After removing the negative urine cultures, we screened the urine culture results from 1959 patients in the baseline period, and from 1958 patients in the intervention period for inclusion (Figure 2). Table 2 shows the baseline characteristics of all included asymptomatic patients and the patients with ASB.

**Table 2.** Baseline characteristics of all included asymptomatic patients.

Characteristics	Baseline group (***n*** = 567)	Intervention group (***n*** = 407)	Total (***n*** = 974)
**Female**	347 (61%)	262 (64%)	609 (63%)
**Median age (IQR)**	74 (62–83)	74 (60–84)	74 (61–83)
**Language barrier**	25 (4%)	17 (4%)	42 (4%)
**Long-term care facility**	36 (6%)	32 (8%)	68 (7%)
**Median CCI (IQR)**	1 (0–2)	1 (0–2)	1 (0–2)
**Indwelling urinary catheter**	60 (11%)	48 (12%)	108 (11%)
**Median MEWS (IQR)**	1 (1–2)	1 (1–2)	1 (1–2)
Treating medical specialty*			
Internal medicine	220 (39%)	138 (34%)	358 (35%)
Geriatrics	37 (7%)	48 (12%)	85 (8%)
Neurology	61 (11%)	32 (8%)	93 (9%)
Surgery	58 (10%)	44 (11%)	102 (10%)
Urology	59 (10%)	51 (13%)	110 (11%)
Emergency medicine	42 (7%)	26 (6%)	68 (7%)
Intensive care	4 (1%)	3 (1%)	7 (1%)
Other	86 (15%)	65 (16%)	151 (15%)
**Admitted to hospital ward**	376 (66%)	261 (64%)	637 (65%)
**Categories of possible infections other than UTIs without antibiotic treatment indication**			
COVID-19	55 (10%)	35 (9%)	90 (9%)
Other viral respiratory tract infection	22 (4%)	18 (4%)	40 (4%)
Gastrointestinal	38 (7%)	39 (10%)	77 (8%)
Skin (Herpes zoster)	7 (1%)	6 (1%)	13 (1%)
Other**	22 (4%)	4 (1%)	26 (3%)

Data are *n* and column percentages may not add up to 100 due to rounding.

* In the Netherlands, physicians of multiple medical specialties work in the ED, therefore, we included these data in the results.

** Viral infections not further described, including viral encephalitis, and yeast or candida infections.

CCI, Charlson Comorbidity Index; ED, emergency department; IQR: interquartile range, MEWS, modified early warning score; UTI, urinary tract infection.

### Primary outcome

In the baseline period, 65/224 (29%) patients with ASB were treated with antibiotics compared to 46/201 (23%) during the intervention period. We observed some variation between the number and percentages of patients with ASB overtreated per hospital during the study period (Supplemental Table 3). The unadjusted fixed effects logistic regression model had the best fit compared to the generalised linear mixed model and the mixed model, and showed no significant reduction of overtreatment of ASB (OR 0.86, 95% 0.40–1.82, *p* = 0.69). To adjust for confounders, we included both age and positive urinalyses results in the model (AIC unadjusted 460.81 vs adjusted 447.17, *p* < 0.001). This model showed no significant effect of the de-implementation strategy in the reduction of overtreatment of ASB (OR 1.20, 95% CI 0.55–2.62, *p* = 0.65).

### Secondary outcomes

#### Asymptomatic patients with contaminated urine cultures

In the baseline period, 18% (102/567) of asymptomatic patients with contaminated urine cultures received antibiotics compared to 15% (62/407) in the intervention period. Both the unadjusted model (OR 0.78, 95% CI 0.43–1.42, *p* = 0.42) and the adjusted model (OR 0.93, 95% CI 0.51–1.69, *p* = 0.81) showed no significant effect.

#### Treatment duration

The mean treatment duration for patients with ASB was more than 2 days shorter in the intervention period (5.79 days, SD 3.33) compared to the baseline period (7.98 days, SD 4.31, mean difference 2.20 days, 95% CI 0.64–3.76). This was also seen in the whole group of asymptomatic patients with contaminated urine cultures (mean difference 1.76 days, 95% CI 0.29–3.23)).

#### The number of urine tests ordered

The number of urinalyses ordered in the ED was reduced from 182 to 153 per 1000 patients. Linear regression analysis results showed an IRD of −29.10 (95% CI −46.36 to −11.78, *p* < 0.001). The reduction from 63 to 56 urine cultures per 1000 patients was not significant (IRD −6.51, 95% CI −14.89 to 1.88, *p* = 0.13).

#### Subgroups

In some specialties, the percentage of overtreatment of all asymptomatic patients with contaminated urine cultures was lower in the intervention period compared to the baseline period (Table 3).

**Table 3.** Asymptomatic patients overtreated with antibiotics by treating medical specialty.

Specialism	Baseline group ***n***/***N*** (%)	Intervention group ***n***/***N*** (%)	Total
**Internal medicine**	37/220 (17%)	11/138 (8%)	48/358 (13%)
**Geriatrics**	10/37 (27%)	11/48 (23%)	21/85 (25%)
**Neurology**	14/61 (23%)	8/32 (25%)	22/93 (24%)
**Surgery**	11/58 (19%)	11/44 (19%)	22/102 (22%)
**Urology**	6/59 (10%)	0/51 (0%)	6/110 (5%)
**Emergency medicine**	12/42 (29%)	5/26 (19%)	17/68 (25%)
**Intensive care**	0/4 (0%)	0/3 (0%)	0/7 (0%)
Other*	12/86 (14%)	16/65 (25%)	28/151 (19%)
**Total**	102/567 (18%)	62/407 (15%)	164/974 (17%)

Data are *n* and row percentages.

* Includes pulmonology, gastroenterology.

n. a., not applicable.

Asymptomatic patients with contaminated urine cultures, who had signs of respiratory tract infections, were more often overtreated than asymptomatic patients with an alternative site (e.g., herpes zoster) of infection for which antibiotics were not indicated (Supplemental Table 4).

#### All patients with positive urinalysis results

During the first study month, 1460 urinalyses were obtained from 8290 adults in all hospitals. We screened 600/1460 (41%) positive urinalyses (Supplemental Figure 2). In total, 436/600 (73%) patients with positive urinalyses had no UTI-related symptoms and were regarded as asymptomatic (Supplemental Table 5). In total, 59/436 (13.5%) asymptomatic patients with positive urinalyses were overtreated with antibiotics, from whom 29 patients had no urine culture obtained.

#### Process and economic evaluation

We did not observe correlations between the number of strategy components in the clusters and the primary outcome (Supplemental Table 6). The results of the screening of the clinical indications for urine testing are listed in Supplemental Table 7.

Based on the IRD of urinalyses ordered in the intervention period, the total hypothetical number of urinalyses would be 8063 instead of 9591 urinalysis. The hypothetical cost savings would be ((8063*€5,53) − (9591*5,53)) = −€8.449,40 for urinalyses in the ED of five hospitals in the baseline period. The combined costs of the de-implementation strategy in five hospitals during the total study period were €2.247,93 (personnel costs: 12 h, which is 0.33 fulltime-equivalent*€6.700,00 = €2.211,00, and study material costs: €36,93). The salary of an antimicrobial stewardship specialist would be similar to that of the primary researcher. Therefore, our de-implementation strategy was cost saving.

## Discussion

After introducing a multifaceted de-implementation strategy, the overtreatment of ASB was not significantly reduced in five EDs in the Netherlands. However, the number of urinalyses ordered in the ED and the treatment duration decreased in the intervention period. These results confirm the importance and relevance of different steps in diagnostic stewardship to reduce the overtreatment of ASB, as shown by the results of previously reported studies that investigated the role of diagnostic stewardship in reducing the overtreatment of ASB.^21–23^

We found a lower percentage of overtreatment of ASB than expected compared to a study that was published before the start of our study,^2^ but similar to studies that were published during our study period.^7,13,24^ Further, during the first study month, we found that 13.5% of asymptomatic patients with positive urinalysis results were inappropriately treated with antibiotics, which was lower than found in a previous study where 24.5% of patients with any degree of pyuria received antibiotics.^25^ This might be explained by the relatively low antibiotic prescription rate in the Netherlands compared to other countries (8.3 vs 18.1 defined daily doses per 1000 inhabitants).^26,27^ Previous studies showed that inappropriate urine testing is associated with prolonged antibiotic treatment.^24,28^ Our de-implementation strategy had a positive effect on appropriate prescribing after the urine culture result was known and emphasised that urine test results should be interpreted with consideration of a clinical assessment.

We focused our de-implementation strategy on the ED and internal medicine specialties, which may explain why the reductions in overtreatment of ASB in these specialties are not reflected in the overall study population. Further, it is possible that the de-implementation strategy needed more time to achieve an effect, which may explain why hospitals 1 and 2 appear to have reduced their overtreatment of ASB in contrast to the other hospitals. Additionally, we hypothesised that a local champion who is also a role model to the other physicians may achieve a greater effect.

A major strength is our study design, allowing us to implement the de-implementation strategy and raise awareness about ASB and its overtreatment in five different types of hospitals with various sizes of patient populations. Further, the adherence to the de-implementation strategy was as high as possible, because the strategies were initiated by the local champions.^29^ To adhere to the work process in EDs as much as possible, we not only focused our de-implementation strategy on ASB but also on all patients with contaminated urine cultures, also those with contaminated and mixed growth as a result, because these were mostly preceded by positive urinalyses.

Our study had some limitations. Firstly, we retrospectively reviewed the management of the urine culture results by evaluating the healthcare professionals’ reports in the electronic patient files. Since this condition was similar in all hospitals in the baseline and intervention periods, we consider the results reliable. Additionally, the appropriateness of clinical indications for antibiotic treatment of positive urine test results was based on the reported UTI-related symptoms as defined by the definitions of ASB and UTIs.^1,16^ Secondly, a common limitation of stepped-wedge cluster randomised trials is that a relatively large sample size and number of clusters are required to achieve an effect. Since no prevalence data were available about ASB and overtreatment in hospitals in the Netherlands, we used a pooled prevalence of 45% (95% CI 34–55) to calculate our sample size.^2^ When we recalculated our sample size with the prevalence in our study, at least 840 patients with ASB were required to achieve sufficient power. This might explain why our reduction in overtreatment of ASB was not statistically significant. Unfortunately, we could not extend the study period for feasibility reasons. Further, we excluded most elderly patients or patients coming from long-term care facilities due to our strict exclusion criteria that consisted of any non-specific infectious disease-related symptoms. Thirdly, patients from whom a urinalysis was obtained during ED presentation were only included during the first study month. Since most clinical decisions in the ED are based on the urinalysis result, the generalizability of our results is limited. Further research is needed to investigate the overtreatment of positive urinalysis results in patients without symptoms of UTIs. Lastly, to account for the clustering and temporal trends, we used fixed effect models to analyse the effect of the de-implementation strategy,^19^ even though alternative models, such as generalised mixed models, would also have been possible. However, the fixed effect model had the best fit.

To improve adequate urine testing, indications for urine testing and interpreting urine test results could be included in the antimicrobial prescribing guidelines. To maintain the effect of the de-implementation strategy and to improve diagnostic stewardship, the hospital’s antimicrobial stewardship team could review the total number and the indications of urine tests ordered on a regular basis. We explored in our process and economic evaluation that appointing an antimicrobial stewardship team specialist is cost saving.

In conclusion, the multifaceted de-implementation strategy to reduce overtreatment of ASB reduced the number of urinalyses ordered and shortened the duration of antibiotic treatment for patients without UTI-related symptoms in the EDs. In EDs, most clinical decisions are based on the urinalysis result. Therefore, multiple steps in diagnostic stewardship are required to improve appropriate urine testing, and subsequently reduce the overtreatment of ASB.

## Supplemental Material

sj-docx-1-tai-10.1177_20499361241293687 – Supplemental material for De-implementation strategy to reduce overtreatment of asymptomatic bacteriuria in the emergency department: a stepped-wedge cluster randomised trial Supplemental material, sj-docx-1-tai-10.1177_20499361241293687 for De-implementation strategy to reduce overtreatment of asymptomatic bacteriuria in the emergency department: a stepped-wedge cluster randomised trial by Tessa M.Z.X.K. van Horrik, Bart J. Laan, Janneke E. Stalenhoef, Cees van Nieuwkoop, Joppe B. Saanen, Caroline Schneeberger, Eefje Jong and Suzanne E. Geerlings in Therapeutic Advances in Infectious Disease

## Declarations

**Ethics approval and consent to participate:** The medical research ethics committee (MREC) “METC Amsterdam UMC” (formerly medical ethics research committee of the Academic Medical Centre, Amsterdam, the Netherlands) confirmed that the Medical Research Involving Human Subjects Act (WMO) does not apply to this study (reference number: W19_472#19.545). Local approval was obtained by the research departments of all participating hospitals. In the Dijklander Hospital and Haga Hospital, an opt-out procedure was followed before collecting data. In the Meander Medical Centre, Amsterdam UMC, and OLVG, a waiver for informed consent was obtained from the local research departments and board of directors, because this project was listed as a quality improvement project and the researchers of the study team were caregivers of the hospital. Therefore, they were allowed to collect data. The trial was registered at https://onderzoekmetmensen.nl/nl/trial/25918. The full study protocol has been published where after we made some adjustments for feasibility reasons (Supplemental Table 1).^17^

**Consent for publication:** Not applicable.

**Competing interests:** BJL received honoraria for giving a lecture at Coloplast BV. JS is chair of the guideline committee for the prevention of catheter-associated urinary tract infections, a member of the guideline committee of the Dutch Association for Urology guideline for urinary tract infections, and received consulting fees from ViiV Healthcare as a member of the HIV advisory board which is unrelated to this manuscript. CS is a board member of the Medical Microbiology Meeting Organization, which is part of the Dutch Association of Medical Microbiology, and for which no payments are received. SEG received funding from the Netherlands Organisation for Healthcare Research and Development for this project, and a consulting fee from Immunotek as a member of the advisory board about a vaccine to prevent urinary tract infections which was paid to the affiliated institution. The other authors declare no competing interests.

**Availability of data and materials:** The de-identified data are available upon publication to investigators whose proposed use of the data has been approved by an independent review committee identified for this purpose. Proposals should be directed to the principal investigator Prof. Dr. Suzanne E. Geerlings (s.e.geerlings@amsterdamumc.nl). To gain access, data requestors will need to sign a data access agreement. The study protocol has been published elsewhere.^17^
