# Supplementary material for: De-implementation strategy to reduce overtreatment of asymptomatic bacteriuria in the emergency department: a stepped-wedge cluster randomised trial
Source: Ther Adv Infect Dis. 2024 Dec 14;11:20499361241293687. doi: 10.1177/20499361241293687 (PMC11645715; doi:10.1177/20499361241293687)
Supplement: sj-docx-1-tai-10.1177_20499361241293687 – Supplemental material for De-implementation strategy to reduce overtreatment of asymptomatic bacteriuria in the emergency department: a stepped-wedge cluster randomised trial [file sj-docx-1-tai-10.1177_20499361241293687.docx]

**Supplementary material belonging to “De-implementation strategy to reduce overtreatment of asymptomatic bacteriuria in the emergency department: a stepped-wedge cluster randomized trial”**

**Supplemental Table 1**. Adjustments to the study protocol

| Item | Clarification of adjustment |
| --- | --- |
| Inclusion criteria  Adult patients with urine culture and/or urinalysis obtained during ED presentation | We included all patients who had urinalyses obtained in all hospitals during the first month. Since the percentage of overtreatment of asymptomatic patients with abnormal urinalyses results without urine cultures was relatively low (13.5%), we discontinued the screening of these patients. After the first month of the study period, we included patents with positive urine cultures, mixed growth, or contaminated cultures (=not negative urine cultures), to focus on the primary endpoint, because most clinical decisions in the ED are based on the urinalysis result. Importantly, the percentage of overtreatment of ASB, a positive urine culture according to the IDSA definition during the first month was almost 40%. |
| Process evaluation  Feedback points for the number of persons present at the planned meetings and for every strategy that is implemented | The hospitals were given points for every strategy that was implemented. However, due to the COVID-19 pandemic measures, some educational meetings were hybrid or through video meetings. Therefore, it was not possible to count the number of persons attending the meetings. |
| Economic evaluation  We will primarily perform a cost-effectiveness analysis, in which we evaluate the cost effectiveness ratio (CER). Our goal was to achieve a reduction in urine culture, urinalysis and antimicrobial prescriptions, and thereby a reduction in costs. (…) The result of the cost-effectiveness analysis will be used in a budget impact analysis to assess the financial impact on the hospital’s budget to implement and sustain the de-implementation strategy | We did not achieve a significant reduction in overtreatment of ASB with antibiotics, but the number of urine tests ordered were reduced. Therefore, we used the numbers of urinalyses and urine cultures ordered to evaluate the cost savings. Data were lacking to perform a full cost-effectiveness analysis, therefore, we performed a budget-impact analysis to estimate the cost-effectiveness of the de-implementation strategy. |

ED: emergency department. ASB: asymptomatic bacteriuria. IDSA: infectious diseases society of America.

**Supplemental Table 2**. The numbers of screened adult patients and incidence rates of urine tests ordered per 1000 adults in each hospital

| Hospital | Adult patients  (N=116282) | Urinalyses  (N=19271) | | Urine cultures  (N=6837) | |
| --- | --- | --- | --- | --- | --- |
|  |  | IR per 1000 adults baseline | IR per 1000 adults intervention | IR per 1000 adults baseline | IR per 1000 adults intervention |
| General teaching hospital 1 | 29109 (25%) | 153 | 145 | 68 | 56 |
| General teaching hospital 2 | 26059 (22%) | 178 | 153 | 70 | 56 |
| Academic hospital 3 | 18259 (16%) | 155 | 127 | 86 | 67 |
| General teaching hospital 4 | 15875 (14%) | 203 | 197 | 41 | 31 |
| General teaching hospital 5 | 26980 (23%) | 188 | 189 | 59 | 60 |

IR: incidence rate

**Supplemental Table 3.** Number and percentages of patients with ASB overtreated with antibiotics per cluster during the study period

| Time period🡪 | 1 | 2 | 3 | 4 | 5 | 6 | Total baseline | Total intervention |
| --- | --- | --- | --- | --- | --- | --- | --- | --- |
| General teaching hospital 1 | 19/40 (47.5%) | 10/30 (33.3%) | 4/18 (22.2%) | 6/21 (28.6%) | 2/17 (11.8%) | 4/21 (19.0%) | 19/40 (47.5%) | 26/107 (24.3%) |
| General teaching hospital 2 | 12/28 (41.9%) | 4/15 (14.8%) | 1/15 (6.7%) | 4/12 (33.3%) | 1/10 (10.0%) | 1/9 (11.1%) | 16/43 (37.2%) | 7/46 (15.2%) |
| Academic hospital 3 | 2/7 (5.4%) | 3/15 (11.1%) | 1/11 (9.1%) | 2/7 (28.6%) | 1/9 (11.1%) | 0/5 (0.0%) | 6/33 (18.2%) | 3/21 (14.3%) |
| General teaching hospital 4 | 0/3 (0.0%) | 1/5 (20.0%) | 1/8 (12.5%) | 3/7 (42.9%) | 2/6 (33.3%) | 3/7 (41.9%) | 5/23 (21.7%) | 5/13 (38.5%) |
| General teaching hospital 5 | 4/19 (17.4%) | 9/25 (36.0%) | 2/12 (16.7%) | 3/14 (21.4%) | 1/11 (9.1%) | 5/14 (35.7%) | 19/85 (22.4%) | 5/14 (35.7%) |

The coloured area represents the intervention period

**Supplemental Table 4**. Patients with signs of possible infections other than UTIs without indications for antibiotics treated for a not-negative urine culture result

| Tract (N) | Baseline  (n=144)  n/N (%) | Intervention  (n=102)  n/N (%) |
| --- | --- | --- |
| COVID-19 (90) | 11/55 (20%) | 5/35 (14%) |
| Other viral respiratory tract infection (40) | 8/22 (36%) | 5/18 (28%) |
| Gastro-intestinal (77) | 4/38 (11%) | 5/39 (13%) |
| Skin (13) | 2/7 (29%) | 2/6 (33%) |
| Other (26) | 0/22 (0%) | 0/4 (0%) |

**Supplemental Table 5.** Baseline characteristics of patients with positive urinalyses regardless of the presence of urine cultures in all hospitals during first study month

|  | | Study month 1  (N=436) | | |
| --- | --- | --- | --- | --- |
| Female | | 301 (69%) | | |
| Median age (IQR) | | 70 (51-81) | | |
| Language barrier | | 26 (6%) | | |
| Long-term care facility | | 20 (5%) | | |
| Median CCI (IQR) | | 0 (0-2) | | |
| Indwelling urinary catheter | | 10 (2%) | | |
| Median MEWS (IQR) | | 1 (1-2) | | |
| Treating medical specialty |  | |  |  |
| Internal medicine | | 123 (28%) | | |
| Geriatrics | | 21 (5%) | | |
| Neurology | | 39 (9%) | | |
| Surgery | | 106 (24%) | | |
| Urology | | 13 (3%) | | |
| Emergency Medicine | | 47 (11%) | | |
| Intensive care | | 7 (2%) | | |
| Other | | 80 (18%) | | |
| Admitted to hospital ward | | 246 (56%) | | |
| Signs of possible infections other than UTIs without indications for antibiotics | | | | |
| No signs of infection | | 325 (75%) | | |
| COVID-19 | | 22 (5%) | | |
| Respiratory tract infection | | 26 (6%) | | |
| Gastrointestinal | | 50 (12%) | | |
| Skin | | 2 (0.5%) | | |
| Other | | 11 (3%) | | |

CCI: Charlson Comorbidity Index; MEWS: modified early warning score; IQR: inter quartile range; data are n and column percentages

**Supplemental Table 6**. Process evaluation of the de-implementation strategy

| Hospital | Local strategy in addition to the general strategy* | Total strategy components |
| --- | --- | --- |
| General teaching hospital 1 | Educational meetings in several departments.  Adjustment of clinical chemistry laboratory orders concerning urinalysis: cancelation of automatic microscopic analysis after a negative urine dipstick result. | 11 |
| General teaching hospital 2 | Educational meetings in several departments.  Adjustment of urine culture orders to cancel automatically ordering urine cultures when a legionella/pneumococcal antigen test is ordered. | 7 |
| Academic hospital 3 | Educational meetings in several departments. | 12 |
| General teaching hospital 4 | Educational meetings in several departments. | 4 |
| General teaching hospital 5 | Educational meetings in several departments. | 7 |

*The general intervention consisted of the following items: a kick-off meeting during which the baseline data was presented as competitive feedback, distribution of pocket cards, posters, sending out newsletters to relevant medical specialties and clinicians working in EDs

**Supplemental Table 7**. Reported clinical indications for ordering urine cultures used for the process evaluation and the de-implementation strategy

|  | Abnormal urine cultures, included in study  (N = 974) | Negative urine cultures, not included in study analyses  (N = 230) | Total (N=1204) |
| --- | --- | --- | --- |
| Inappropriate indications  (no reported indication, antigen test, no UTI-related symptoms, gastrointestinal symptoms, on request) | 431 (44%) | 73 (32%) | 504 (42%) |
| Appropriate indications  (UTI-related symptoms, urological symptoms) | 123 (13%) | 52 (23%) | 175 (15%) |
| Indications that could be both appropriate and inappropriate  (non-specific urogenital symptoms, signs of systemic infection, recent use of indwelling catheter, recent UTI, catheter-related problems, e.g., obstruction) | 420 (43%) | 105 (46%) | 525 (44%) |

Data are n and column percentages; percentages may not add up to 100 due to rounding. For most patients, multiple symptoms or indications were reported. If at least one reported symptom or indication was considered appropriate, we categorised this indication as ‘appropriate’. If none of the reported symptoms or indications was considered appropriate, we categorised this indication as ‘inappropriate’.

**Supplemental Figure 1**. Study materials used in the de-implementation strategy


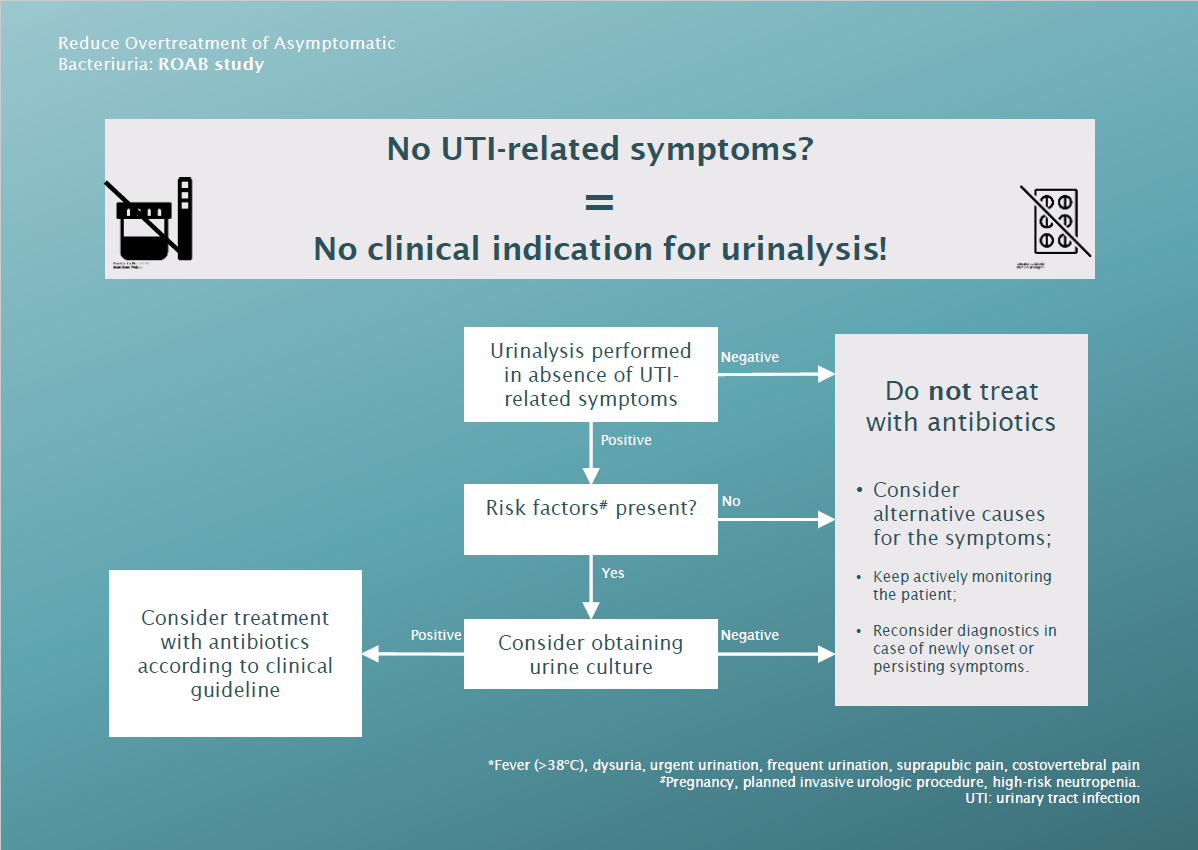


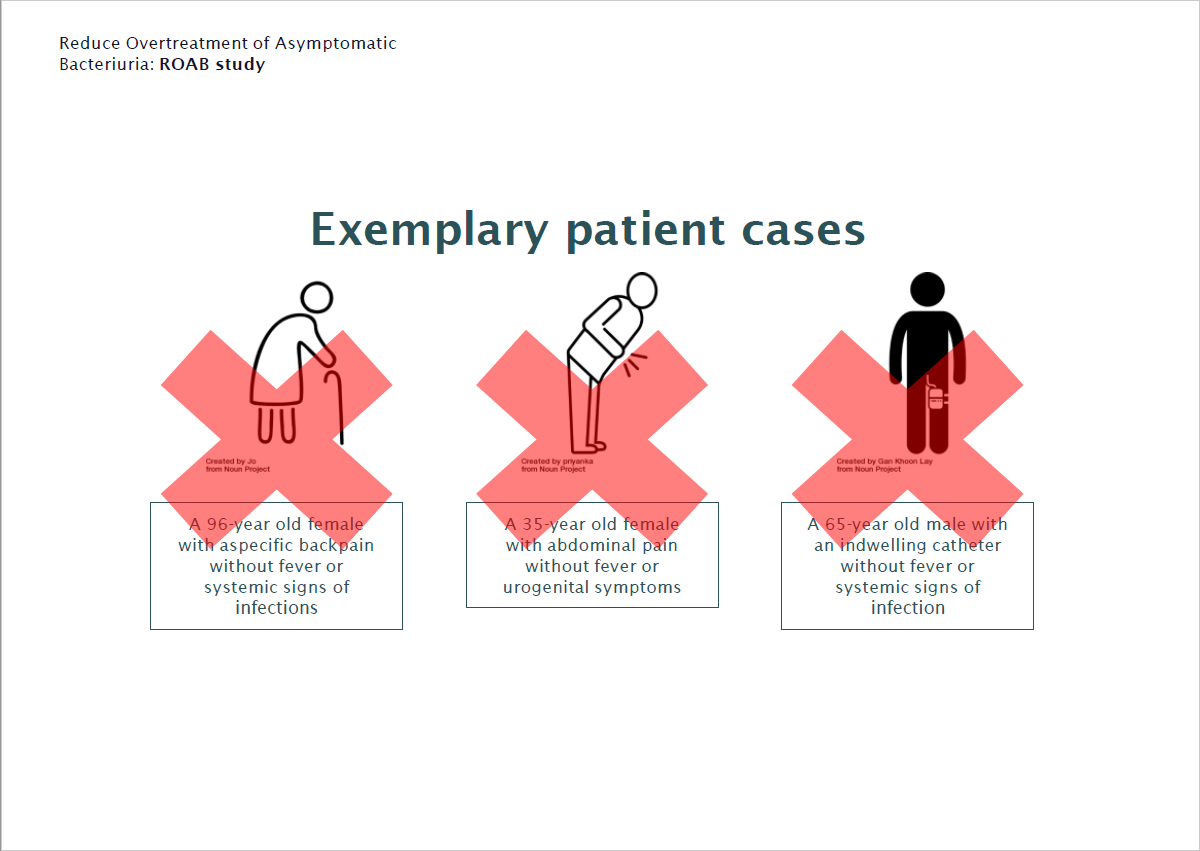


**Supplemental Figure 2**. Flowchart of asymptomatic patients with positive urinalyses during the first study month


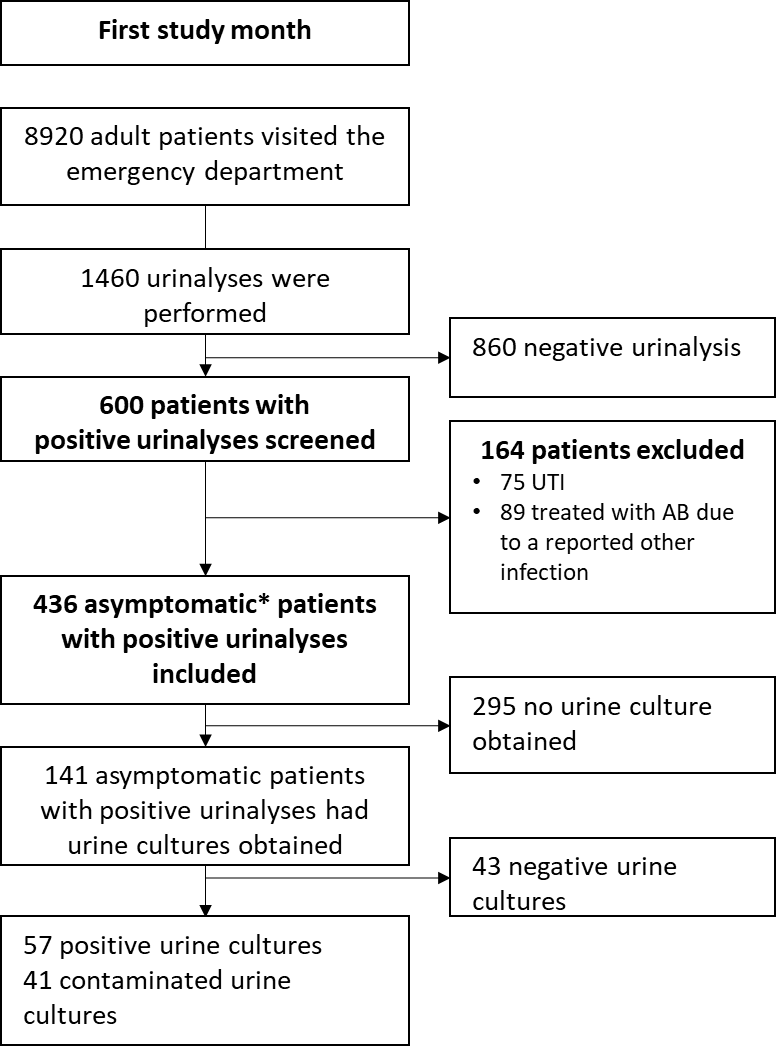


*patients without UTI-related symptoms, including patients with possible signs of infections other than UTIs without clinical indications for antibiotic treatment. AB: antibiotics; UTI: urinary tract infection
